# Real time monitoring of aminothiol level in blood using a near-infrared dye assisted deep tissue fluorescence and photoacoustic bimodal imaging†

**DOI:** 10.1039/c5sc04986e

**Published:** 2016-03-02

**Authors:** Palapuravan Anees, James Joseph, Sivaramapanicker Sreejith, Nishanth Venugopal Menon, Yuejun Kang, Sidney Wing-Kwong Yu, Ayyappanpillai Ajayaghosh, Yanli Zhao

**Affiliations:** a Chemical Sciences and Technology Division and Academy of Scientific and Innovative Research (AcSIR), CSIR-National Institute for Interdisciplinary Science and Technology (CSIR-NIIST) Thiruvananthapuram 695019 India ajayaghosh@niist.res.in; b Division of Chemistry and Biological Chemistry, School of Physical and Mathematical Sciences, Nanyang Technological University 21 Nanyang Link 637371 Singapore zhaoyanli@ntu.edu.sg; c School of Chemical and Biomedical Engineering, Nanyang Technological University 62 Nanyang Drive 637459 Singapore; d Faculty of Materials and Energy, Southwest University 2 Tiansheng Road Chongqing 400715 P. R. China; e Department of Nuclear Medicine & PET, Singapore General Hospital Outram Road 169608 Singapore; f School of Materials Science and Engineering, Nanyang Technological University 639798 Singapore

## Abstract

The development of molecular probes for the detection and imaging of biological thiols is a major step forward diagnosing various types of diseases. Previously reported thiol imaging strategies were mainly based on a single mode of imaging with a limited application *in vivo*. In this work, we introduced an unsymmetrical near-infrared (NIR) squaraine dye (USq) as an exogenous contrast agent for photoacoustic and fluorescence bimodal imaging of thiol variations in live animals. USq exhibits a narrow absorption band at 680 nm that generates a photoacoustic signal and a strong NIR emission at 700 nm (*Φ*_F_ = 0.27), which is applicable for deep tissue optical imaging. Both photoacoustic and fluorescence signals could selectively disappear in the presence of different thiols. Through *in vitro* and *in vivo* imaging studies, unique imaging capability of USq was demonstrated, and the effect of food uptake on the increased level of aminothiols in blood was confirmed.

## Abstract 2

Real time monitoring of blood aminothiol level by a near-IR dye assisted fluorescence–photoacoustic bimodal imaging is reported.

## Graphical abstract

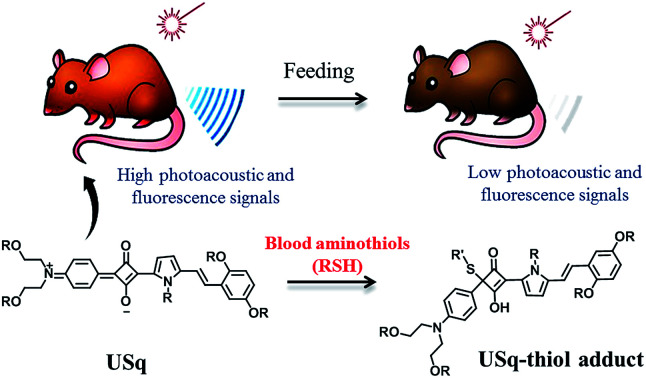

## Introduction

Cellular thiols are essential biomolecules that play a major role in maintaining various biological processes, including redox homeostasis and cellular growth.^1^ Abnormal levels of these biomolecules are closely associated with certain disease states, including liver damage, cancer, AIDS, Alzheimer's disease, and cardiovascular diseases.^2^ Therefore, monitoring the level of thiol-containing substances in biological systems may facilitate early diagnosis and prevention of these diseases. Recently, considerable effort has been devoted to the development of novel techniques that could perform high resolution *ex vivo* or *in vitro* monitoring of aminothiols.^3^ However, non-invasive imaging of aminothiols in living organisms still remains challenging. Amongst various imaging techniques used in current pre-clinical and clinical settings, optical techniques are more favored for aminothiol detection due to their high sensitivity, operational simplicity and low costs.^4–6^ Often these techniques are used in conjunction with aminothiol-sensitive molecular probes that bind to a specified target site. In recent years, there has been remarkable progress in establishing optically activatable probes for visualizing biological thiols in living systems.^7^ However, the usage of the UV absorbing probes in live subjects is often challenged due to autofluorescence and high levels of light extinction in tissues. Thus, the lack of autofluorescence and the increased penetration depth across near-infrared (NIR) region (650–950 nm) make fluorescent probes with NIR absorption and emission more suitable for *in vivo* bioimaging applications.^8^ In this context, aminothiol-sensitive NIR absorbing probes are of great importance.^9^

Considering various preclinical optical imaging techniques, multispectral optoacoustic tomography (MSOT) with unique imaging capability has shown promising results in overcoming the penetration depth and spatial resolution limits of conventional optical imaging modalities.^10^ MSOT is a non-invasive optical imaging modality based on the principles of the photoacoustic effect. This technique involves the reconstruction of images from ultrasound waves generated due to thermoelastic expansion of molecules upon selective photon absorption.^11^ MSOT when coupled with multispectral NIR excitation provides high contrast, deep tissue multispectral imaging capability with high spatial resolution (∼150 μm) and penetration depths (>5 cm). It is therefore expected that the synthesis of aminothiol sensitive probes that exhibit good NIR absorption could enable deep tissue visualization of aminothiols *in vivo*. To the best of our knowledge, no study has been reported so far to demonstrate high resolution, deep tissue optical imaging of thiols using MSOT and NIR fluorescence bimodal approach.

**Scheme 1:**
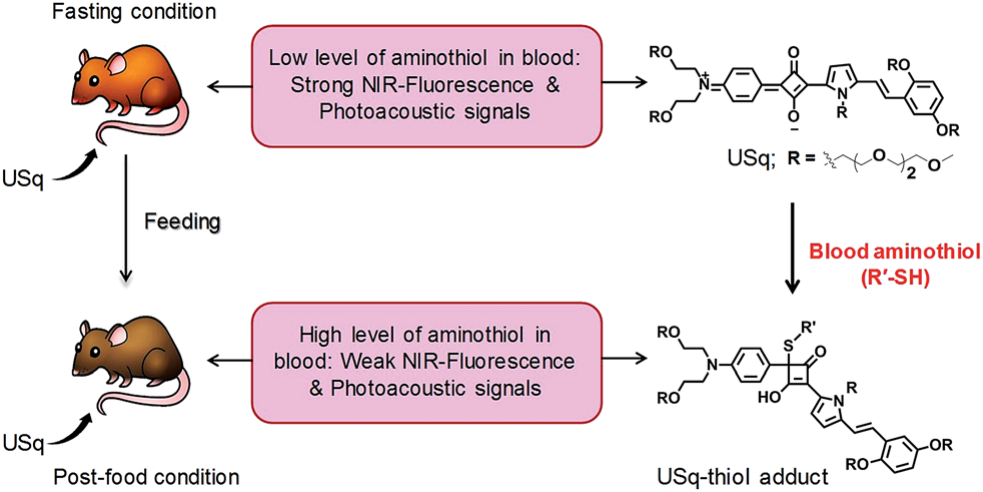
Schematic diagram depicting the realization of fluorescence and photoacoustic bimodal signaling of aminothiol content *in vivo* using USq.

Squaraines are an important class of NIR dyes, which are extensively used in a wide range of imaging applications.^12^ Herein, we report the experimental demonstration of an aminothiol sensitive NIR squaraine probe (USq) for fluorescence and MSOT imaging applications *in vivo* (Scheme 1). Effective use of a squaraine probe for *in vivo* applications relies on preventing its aggregation in aqueous conditions.^13,14^ For this reason, we synthesized an unsymmetrical squaraine dye decorated with triethylene glycol chains that provide solubility to the dye and thereby prevents its aggregation under biological conditions.^15^ In aqueous conditions, USq exhibited excellent solubility, high chemical stability and low cytotoxicity. Upon reaction with aminothiols, NIR to visible ratiometric fluorescence changes were observed in analogy to the previous reports.^16^ The promising potential of USq for fluorescence and photoacoustic bimodal signaling of aminothiol content *in vivo* was demonstrated.

## Results and discussion

**Fig. 1:**
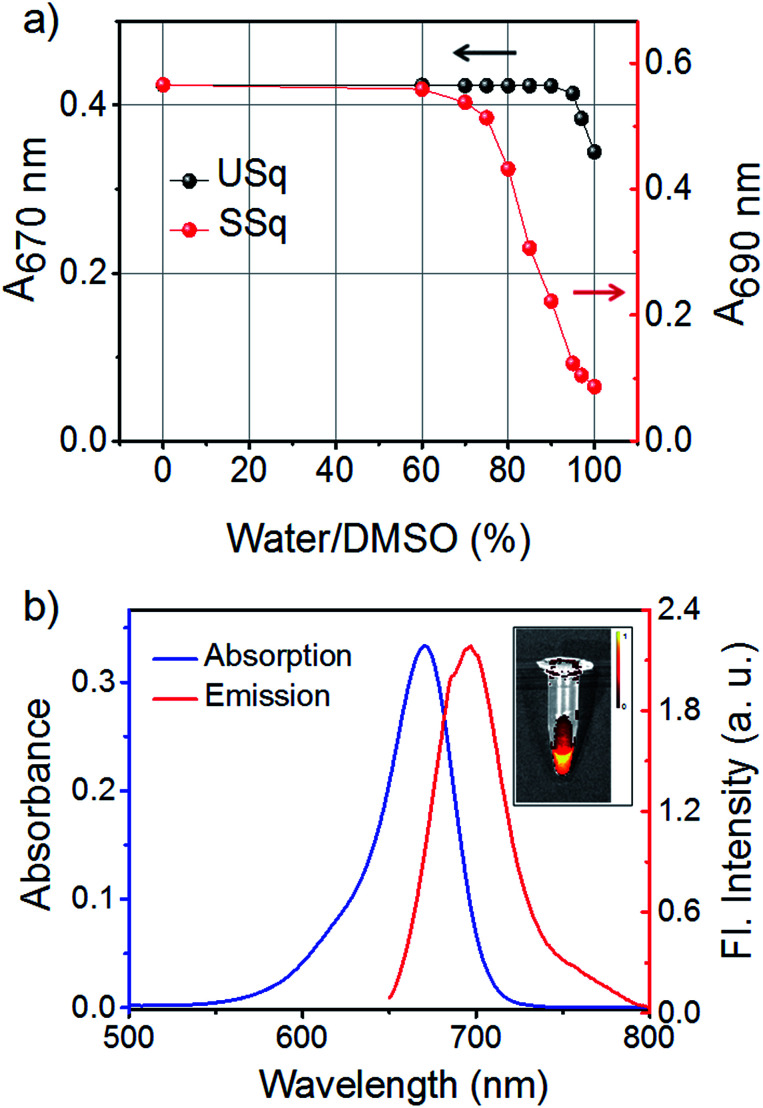
(a) Changes in the absorption spectra of USq and SSq (2 μM) in DMSO upon increasing the content of water from 0–100%. (b) UV/Vis absorption (blue curve) and emission spectra (red curve, *λ*_ex_ = 640 nm) of USq (2 μM) in 96% phosphate buffer (pH 7.8)/DMSO mixture. Inset shows false-color pixel intensity map image of USq (*λ*_ex_ = 640 nm) in 96% phosphate buffer/DMSO mixture.

The NIR absorbing squaraine dye USq was synthesized according to the standard procedure as described in the ESI.† The dye was purified by column chromatography and characterized by NMR and mass spectrometry. UV/Vis spectrum of USq (2 μM) in dimethyl sulfoxide (DMSO) displayed a narrow and intense absorption peak with the maximum at 680 nm (*ε* = 1.94 × 10^5^ L mol^−1^ cm^−1^), and it showed the emission at 705 nm (*Φ*_F_ = 0.27 with Nile blue in ethanol as reference *Φ*_R_ = 0.27) (Fig. S1a and b†). The inset of Fig. S1b† shows a false-color pixel intensity map for the fluorescence of USq (*λ*_ex_ = 640 nm). Aqueous compatibility of USq was tested in different water/DMSO solvent mixtures. Fig. 1a shows the variation of absorption maximum of USq (2 μM) upon changing the water content. The absorption spectra indicate that the dye did not aggregate in up to 96% water/DMSO mixture. The absorption and emission spectra of USq in 96% phosphate buffer/DMSO mixture is shown in Fig. 1b. The inset exhibits the corresponding false color pixel intensity image upon excitation at 640 nm. A comparison of solubility between USq and a model symmetric squaraine dye (SSq, see ESI† for chemical structure) indicates high solubility of the former in water to prevent the dye aggregation, which is beneficial to the interaction with thiols at cellular condition.

Initially, we investigated the photophysical changes of USq upon the addition of thiol-containing biorelevant molecules such as glutathione (GSH). Fig. S2† shows the UV/Vis absorption and fluorescence spectral changes upon the addition of GSH to USq (2 μM) in 96% phosphate buffer (pH 7.8)/DMSO mixture. Titration of GSH with USq induces a decrease in the absorption maximum at 670 nm with a concomitant formation of a new band at 380 nm (Fig. S2a†). Similarly, USq displayed a new emission band at 520 nm (*λ*_ex_ = 380 nm) with simultaneous quenching of the 700 nm emission upon the addition of GSH (Fig. S2b and c†). These changes in the absorption and emission spectra are due to the addition of the thiol group to the cyclobutene ring of the squaraine moiety, resulting in the activation of a strongly fluorescent dormant fluorophore.^14*b*^ The insets of Fig. S2b and c† show the false color pixel intensity mapping to visualize the emission changes upon excitation at 430 and 640 nm, respectively.

**Fig. 2:**
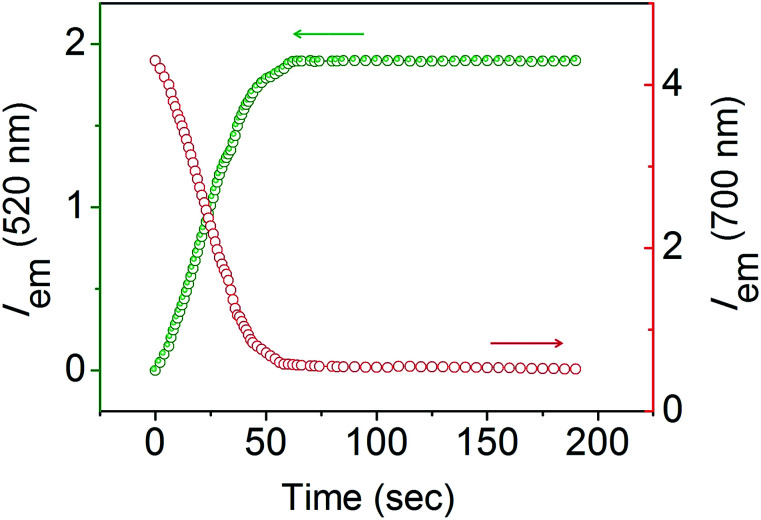
Fluorescence intensity changes at 520 (*λ*_ex_ = 380 nm) and 700 nm (*λ*_ex_ = 640 nm) produced by the reaction of USq (2 μM, in 96% phosphate buffer (pH 7.8)/DMSO mixture) with GSH (20 μM). The reaction was continuously monitored at time interval of 1 s.

The response velocity of USq toward GSH was examined by monitoring the fluorescence intensity at 520 and 700 nm as a function of time (Fig. 2). The addition of 10 equiv. of GSH resulted in an enhancement in the fluorescence intensity at 520 nm with simultaneous quenching of the 700 nm emission. These changes in the fluorescence intensities were saturated within 2 min, implying the fast response of the probe to GSH. Furthermore, USq can detect GSH even at 5 nM level with the fluorescence intensity, exhibiting a good linearity with the concentration of GSH (0–50 nM, *R* = 0.934, Fig. S3†). These results indicate that USq exhibits a fast response to thiol with an ultra-low detection limit.

According to the aforementioned speculation, the thiol group in GSH can undergo a nucleophilic addition at the double bond that is proximate to the cyclobutene ring in USq. When USq is in its resonance stabilized structure, this double bond is delocalized near to aniline or pyrrole ring, and the reaction preferentially occurs on one of these electrophilic centers. If the reaction occurs near the aniline ring, it follows pathway 1 by evoking semi-squaraine of the styryl-pyrrole chromophore as depicted in Fig. S4a.† Conversely, if it happens near to the pyrrole ring, only styryl-pyrrole chromophore becomes free as shown in pathway 2 (Fig. S4a†). In order to understand the actual reaction site, we compared the fluorescence spectra of the thiol adduct with free styryl-pyrrole chromophore and its semi-squaraine (Fig. S4b†). Emission spectrum of the thiol adduct exhibited a peak at 520 nm that matches well with the spectral profile of the semi-squaraine derivative, implying that the actual reaction mechanism occurs through pathway 1 (Fig. S4c†).

Since there is an increasing demand for real-time monitoring of GSH during biochemical processes, the construction of functional approaches using small molecule based fluorescent probes that could quickly and reversibly recognize and detect the GSH analyte is of current interest. However, fluorescent probes based on small organic molecules interacting with the thiol moiety (–SH) of the analyte are generally irreversible and are not fast enough to allow real-time detection under physiological conditions. It is expected that the unique photophysical properties of USq could offer potential capabilities to overcome these issues. The addition of a competing ligand, *N*-ethyl maleimide (NEM), to a solution of USq–GSH adduct could release the dye molecule and make it free for further sensing of GSH (Fig. S5†). The fluorescent intensity of USq–GSH adduct at 520 nm decreases with concomitant formation of 700 nm emission upon the addition of NEM (Fig. S5b†). Further addition of GSH to this solution again reverses the process. This dynamic nature of the bond offers reversible interconversion of USq–GSH interaction, which can be repeated for several cycles (Fig. S5c†), demonstrating the capability of real time monitoring of thiols in biological systems.

Next, we investigated the selectivity of USq toward other biologically relevant amino acids under the same experimental conditions. Amongst the tested analytes (Fig. S6†), GSH, cysteine (Cys) and homocysteine (Hcy) exhibited the highest fluorescence response. Moreover, USq showed a similar fluorescence change with GSH in the presence of these competing amino acids, indicating that the probe offers a high selectivity for thiol-containing biomolecules without interference from other biomolecules. Furthermore, the pH dependence of thiol-mediated nucleophilic addition reaction was also investigated. No variation in fluorescence emission at 700 nm was observed for the free dye over a wide pH range (Fig. S7†), indicating the stability of USq across the pH range of 4–10. On the other hand, it readily reacts with GSH within the biologically relevant pH range (6–9). These features also indicate that USq could be effectively used to detect the presence of cellular thiols without any interference from pH effects. These prominent features of this ratiometric probe prompted us to further examine its suitability for visualizing endogenous thiols in live cells and animals.

**Fig. 3:**
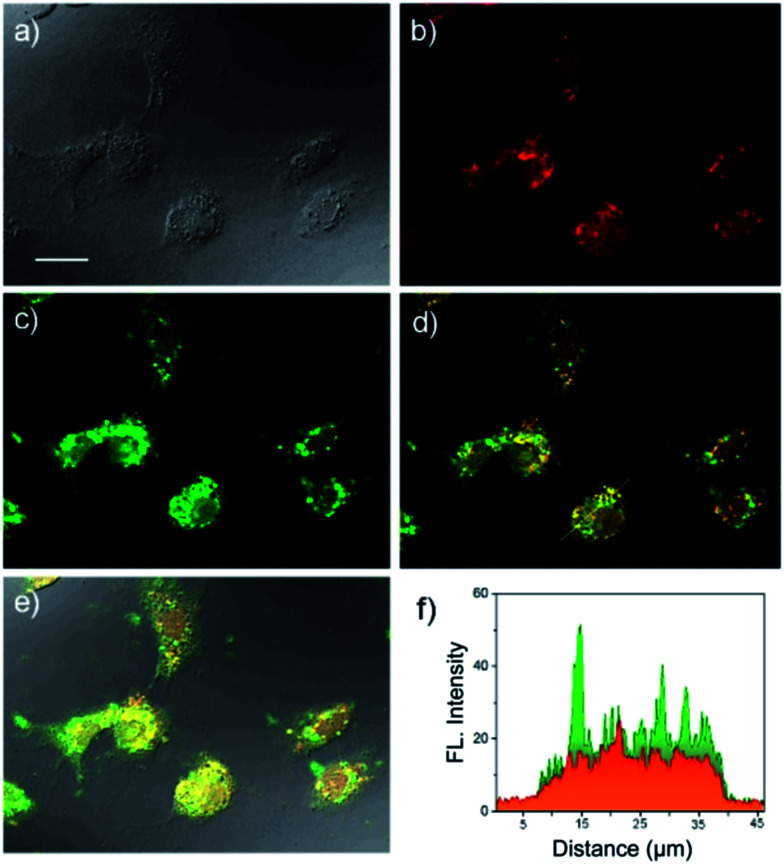
Fluorescence microscope images of Huh-7 cell line incubated with USq for 12 h. (a) Bright field image (scale bar = 25 μm), (b) fluorescence image, *λ*_ex_ at 620 nm, *λ*_em_ collected at 650–750 nm, (c) fluorescence image, *λ*_ex_ at 405 nm, *λ*_em_ collected at 480–600 nm, (d) image obtained by overlay of (b) and (c), (e) image obtained by overlay of (a–c), and (f) fluorescence intensity profiles at 680 nm (red) and 520 nm (green) through the dotted line as indicated in image (d).

The *in vitro* fluorescence imaging capability of USq and its ability to respond to biological thiols inside cells were examined by fluorescence microscopic studies in human hepatoma cell line (Huh-7 cell lines). Huh-7 cells were incubated with USq for 12 h, and fluorescence microscopy images were obtained at 620 nm excitation (*λ*_em_ collected: 650–750 nm) and 405 nm excitation (*λ*_em_ collected: 480–600 nm), respectively. As shown in Fig. 3c, a strong green fluorescence upon excitation at 405 nm was observed inside the cells, indicating the internalization of USq and its conjugation with cellular thiols. On the other hand, excitation at 620 nm gave a red fluorescence with weak intensity (Fig. 3b). The *in vitro* thiol distribution was evident from the fluorescence overlay images in Fig. 3d and e, where green dominated region indicates the USq–thiol adduct, while the yellow region indicates the presence of free dye as well as the adduct. The fluorescence intensity profile (Fig. 3f) shows relative intensities of USq and USq–thiol adduct *in vitro*. Together with the low cytotoxicity shown by 3-(4,5-dimethylthiazol-2-yl)-2,5-diphenyltetrazolium bromide (MTT) assay (Fig. S8†) and the *in vitro* fluorescence experiments, the promising potential of USq for *in vitro* thiol imaging was successfully demonstrated.

**Fig. 4:**
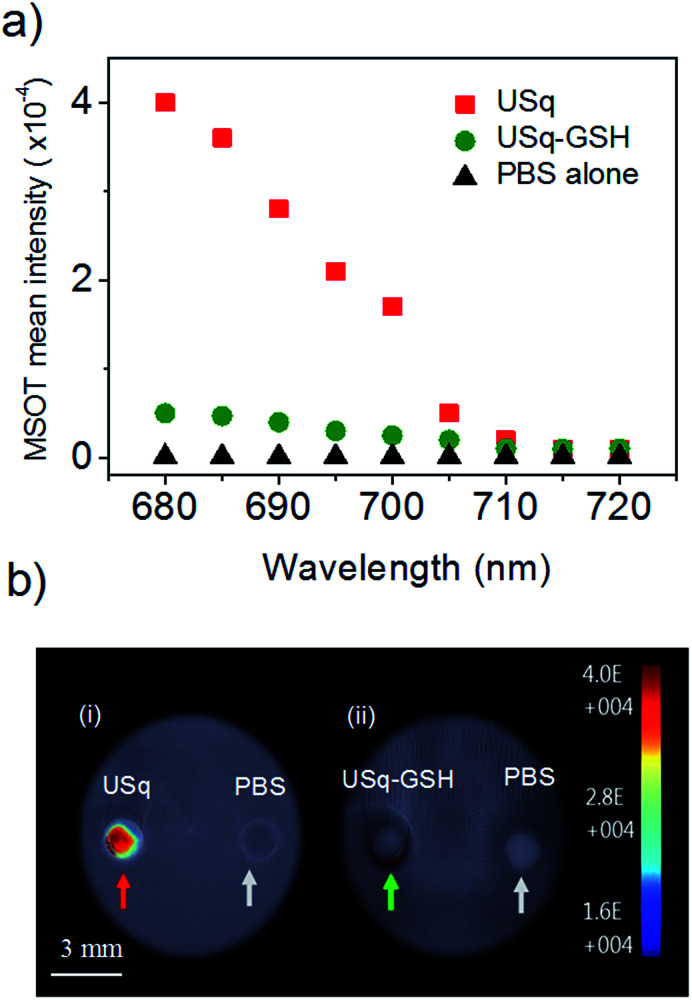
(a) MSOT mean intensity of USq (red squares) and USq–GSH adduct (green circles) in comparison with control (PBS buffer at pH 7.4, black triangles). (b) Single wavelength photoacoustic images of phantom acquired at 680 nm. Red and green arrows indicate channels containing (i) USq and (ii) USq–GSH adduct with respect to blank (PBS buffer).

Prior to *in vivo* photoacoustic imaging studies, photoacoustic signal generation from USq was studied using a cylindrical tissue-mimicking phantom. As evident from Fig. 4a and b(i), USq exhibits excellent photoacoustic response due to significant absorption at 680 nm, thereby demonstrating its photoacoustic imaging capabilities. However, due to negligible absorption at 680 nm, the USq–GSH adduct failed to generate sufficient photoacoustic signals as shown in Fig. 4b(ii).

**Fig. 5:**
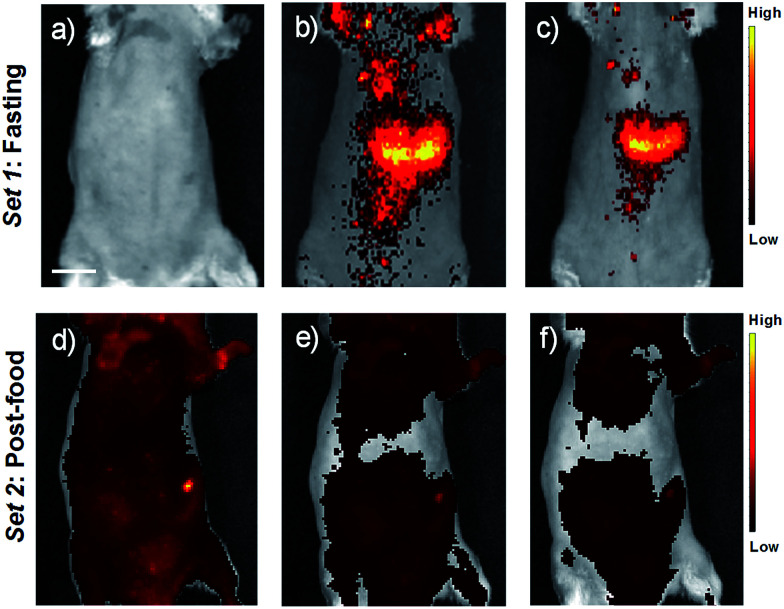
*In vivo* fluorescence reflectance images obtained after injecting with USq in fasting mouse (Set 1) and post-food mouse (Set 2). Fluorescence reflectance image from dorsal side of (a) pre-injected with USq in a fasting female mouse at 675 ± 15 nm excitation and 700 ± 10 nm emission. (b and c) Images recorded after 15 min and 40 min of USq injection in fasting condition, respectively. Images of mouse in post-food condition recorded upon excitation at 675 ± 15 nm excitation and 700 ± 10 nm emission, and images were recorded at (d) 5, (e) 15 and (f) 40 min intervals. Scale bar = 1.5 mm.

For *in vivo* imaging studies (both fluorescence and photoacoustic modes), seven-week-old severe combined immune deficiency (SCID) female mice under fasting conditions were initially imaged. Upon the recovery, the animals were fed and then knocked down for performing post-food imaging. Fluorescence imaging of the animals was carried out first, and immediately followed by photoacoustic imaging of the same animal. The animals were anaesthetized and maintained under 2% isoflurane throughout the experiments. Prior to imaging, belly furs of the animals were removed using a razor and depilatory cream. USq (2 μM, 200 μL) was administered intravenously through the tail vein for fluorescence and photoacoustic imaging studies. Fluorescence imaging of the mice was carried out with the mice placed in supine position. Fig. 5a shows fluorescence images of a fasting mouse prior to injection with USq. No fluorescence signals were detectable from the mouse body at 675 ± 15 nm excitation and 700 ± 10 nm emission settings. Fig. 5b and c show images recorded 15 and 40 min post-injection of USq, respectively. A strong red fluorescence emission from the mouse indicates effective distribution of free dye in blood and its accumulation in the abdominal region. Fig. 5d–f show the images of the mouse in the post food condition after being administered with USq. Here, the fluorescence signal intensities were weak, indicating that after feeding, the thiol production inside the body was high and as-generated thiols were reacted with USq.

**Fig. 6:**
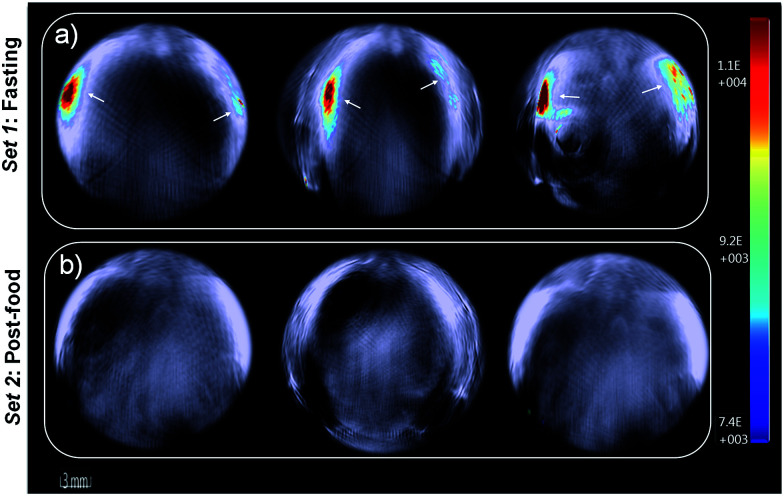
Single wavelength MSOT images of live mouse anatomy at 680 nm. (a) Individual anatomy sections of fasting mouse at 40 min post-injection of USq. (b) Anatomy sections of post-food mouse at 40 min post injection of USq. Negligible signals indicate the formation of USq–thiol adduct *in vivo*.

Furthermore, *in vivo* photoacoustic imaging of biological thiols in blood was carried out in the same mice before and after feeding. For *in vivo* multispectral optoacoustic imaging, we used an excitation wavelength range from 680 to 720 nm with 5 nm step intervals, together with 730, 800 and 850 nm excitations. Photoacoustic signals were acquired with 10 times frame averaging for each excitation wavelength. Machine integrated model-based reconstruction approach was utilized to generate photoacoustic images, and signals from USq were spectrally resolved using a linear regression based spectral unmixing approach. Images acquired from the fasting mice and post-feed mice were analyzed by drawing the region of interest (ROI) on the liver slice. Fig. 6 shows spectrally resolved photoacoustic images of the abdominal region of a female mouse excited at 680 nm. Set 1 images obtained from the fasting mouse clearly indicate the accumulation of USq in the abdominal region (arrows indicate intense signals from USq *in vivo*). Set 2 images were acquired from the same mouse with post-food after injecting with USq.

Signal intensity variations of USq injected mice in fasting and post-food conditions clearly showed the differences in both fluorescence and photoacoustic signal intensities. An increased level of aminothiol concentration in blood under post-food condition is evident from the experimental observations through the formation of a USq–thiol adduct. Thus, the bimodal experimental confirmation of the present strategy is expected to provide scientific clues toward the prediction and treatment of aminothiol contents in patients suffering from related diseases using non-invasive optical imaging techniques. Precise quantification of aminothiol level is possible upon the extension of the current bimodal approach and will be highly useful for early prediction, diagnosis and treatment of coronary heart diseases, atherosclerosis, and post-radiation syndromes.

## Conclusions

We have demonstrated the application of an unsymmetrical squaraine dye, USq, for real time *in vivo* and *in situ* fluorescence and photoacoustic bimodal signaling of aminothiols in living animals. USq exhibited excellent solubility in aqueous conditions. Since it did not form aggregates in aqueous condition, it exhibited intense absorption with a maximum peak at 670 nm and strong fluorescence. These signals could selectively disappear in the presence of thiols. The excellent cell permeability, low cytotoxicity and high stability in aqueous conditions inspired us to explore the dye for real time imaging of thiol variations in living systems. We have utilized this probe for establishing a known metabolic activity of modulating the thiol production by altering the food intake. The experiments performed with live mice in fasting and post-food conditions well matched with the expectations, implying the versatility of this probe in understanding the metabolic activity of a living system. The results presented here demonstrate promising potential of USq in diagnosing various types of diseases that are related to thiol variations.

## Experimental

### Materials and methods

All chemical reagents, unless otherwise specified, were purchased from Sigma-Aldrich Co. All solvents were of reagent grade and were purchased from local companies. All solvents were dried and distilled prior to use by following standard procedures. ^1^H NMR spectra were recorded on a Bruker 500 MHz FT-NMR (Advance-DPX 300) spectrometer at 25 °C. The chemical shift (*δ*) and coupling constant (*J*) values were given in parts per million (ppm) and hertz (Hz), respectively. Matrix-assisted laser desorption/ionization time-of-flight (MALDI-TOF) mass spectra were obtained on an AXIMA-CFR PLUS (SHIMADZU) spectrometer using α-cyano-4-hydroxycinnamic acid as a matrix. High-resolution mass spectra (HRMS-FAB) were recorded on JEOL JM AX 505 HA mass spectrometer. UV/Vis spectra were obtained by using a Shimadzu UV-3600 UV/Vis-NIR spectrometer. Steady-state emission spectra at room temperature were obtained using a Shimadzu RF-5301PC spectrofluorimeter. *In vivo* fluorescence imaging studies were performed using an IVIS lumina II preclinical imaging system and analyzed using the IVIS Living Imaging 4.4 software (PerkinElmer Inc., Alameda, CA, USA). Photoacoustic imaging studies of the tissue mimicking phantom and live mice were performed using the MSOT system (MSOT inSight 64, iThera Medical GmbH).

### *In vitro* cytotoxicity assay

MTT assay was carried out by following standard procedures. Huh-7 cells were seeded into a 96-well plate (1 × 10^4^ cells per well) in DMEM (Dulbecco's Modified Eagle's Medium) cell culture medium containing 10% fetal bovine serum and grown under a humidified atmosphere with 5% CO_2_ at 37 °C. After 12 h incubation, the media in the wells were replaced with fresh DMEM (100 μL per well) containing USq with different concentrations, and the cells were further incubated for 12 h. Then, the medium was changed by DMEM (100 μL per well) containing MTT (0.5 mg mL^−1^), followed by incubation for another 4 h. The culture medium was removed and frozen crystals were dissolved with freshly prepared DMSO (100 μL). Before the cytotoxicity measurement, the plate was agitated gently for 15 min, and then the absorbance intensity at 560 nm was recorded by a micro plate reader. The cell viability (%) for each sample relative to the control well was finally calculated.

### *In vivo* fluorescence imaging

*In vivo* imaging studies were carried out using seven-week-old SCID female mice (*n* = 3). All the *in vivo* studies were conformed to the Guide for the Care and Use of Laboratory Animals published by the National Institutes of Health (NIH), USA and protocol approved by the Institutional Animal Care and Use Committee (IACUC) National University of Singapore. Belly fur of the mice was removed using the depilatory cream prior to any imaging procedure. *In vivo* fluorescence imaging studies of the live mice were performed using an IVIS spectrum preclinical imaging system and analyzed using the IVIS Living Imaging 4.4 software. A back-thinned back-illuminated CCD camera having 2048 × 2048 pixels cooled to −90 °C was used as the detector. Similar illumination and acquisition settings were used for acquiring all the fluorescence images. Images were acquired with binning factor 8, 4 s exposure time, and 12.9 cm field of view. Mice were placed on a temperature-controlled stage inside the imaging chamber of the system that was equipped with an integrated isoflurane based anaesthesia system.

It was well established that the total aminothiol content in blood plasma could vary due to many reasons, and aminothiol levels in blood plasma could be modulated by altering the food intake.^17^ Therefore, two distinct experimental conditions were adopted for the *in vivo* imaging studies. The first experimental condition was introduced to minimize aminothiol levels in the blood plasma. For this purpose, the mice were scheduled to fast for 6 h prior to the *in vivo* imaging studies, which would result in the reduction of aminothiol levels in blood. The second experimental condition for *in vivo* imaging studies was performed after feeding the animals. This condition induces methionine loading that contributes to increased levels of aminothiols in blood. Each set of experiments was repeated for three times independently.

### Multispectral optoacoustic imaging studies

Photoacoustic imaging studies of tissue mimicking phantom and live mice were performed using the MSOT system. The imaging system consists of a tunable nanosecond pulsed laser, a spherical array ultrasound detector, a parallel data acquisition system and a computer. The optical parametric oscillator based tunable laser generating 5 ns duration pulses having energy of 20 mJ and repetition rate of 10 Hz serves as the excitation source. The output of the laser source could be tuned from 680 to 980 nm per pulse basis. The output beam of the laser is coupled into a fiber bundle to enable excitation beam uniformly delivered onto the surface of the sample. Photoacoustic signals were detected using a 64-element piezoelectric transducer array having a spherical geometry, which can provide a tomographic view of 172°.

Photoacoustic signal generation from USq was first studied using a cylindrical shaped tissue-mimicking phantom (iThera Medical GmbH). The phantom having 2 cm diameter had similar optical and acoustic properties as that of biological tissues. USq in PBS buffer at pH 7.4 was loaded into one of the two sample channels located inside the phantom, and same volume of control (PBS buffer at pH 7.4) was loaded into the second channel. Photoacoustic signals were then measured under excitation wavelengths ranging from 680 to 720 nm at 5 nm step intervals and were acquired using the 64-element ultrasound transducer array. Multispectral photoacoustic images were generated with 25 times frame averaging per wavelength and processed using standard machine integrated viewMSOT software.

By following previously explained anaesthesia procedure, the animal was anaesthetized and placed in supine position over a thin polyethylene membrane. The polyethylene membrane prevents direct contact of the mouse with water and permits excellent acoustic coupling between mouse and detector array. The animal was then placed inside the water filled imaging chamber using the supplied animal holder. The water bath was temperature controlled, and the mouse holder could be translated across the imaging plane to obtain multiple transverse image slices of the mouse. Photoacoustic images were acquired with an in-plane resolution of approximately 180 μm. Each set of experiments was repeated for three times independently.

## Supplementary Material

SC-007-C5SC04986E-s001
