# Supplementary material for: Real time monitoring of aminothiol level in blood using a near-infrared dye assisted deep tissue fluorescence and photoacoustic bimodal imaging
Source: Chem Sci. 2016 Mar 2;7(7):4110–6. doi: 10.1039/c5sc04986e (PMC6013924; doi:10.1039/c5sc04986e)
Supplement: SC-007-C5SC04986E-s001 [file SC-007-C5SC04986E-s001.pdf]

Electronic Supplementary Material (ESI) for Chemical Science.
This journal is © The Royal Society of Chemistry 2016

Electronic Supplementary Information (ESI)

Real Time Monitoring of Aminothiol Level in Blood Using a Near-Infrared Dye Assisted Deep Tissue Fluorescence and Photoacoustic Bimodal Imaging

Palapuravan Anees,[a,§] James Joseph,[b,§] Sivaramapanicker Sreejith,[b,§] Nishanth Venugopal Menon,[c] Yuejun Kang,[c,d] Sidney Wing-Kwong Yu,[e] Ayyappanpillai Ajayaghosh*,[a] and Yanli Zhao*,[b,f]

[a]Chemical Sciences and Technology Division and Academy of Scientific and Innovative Research (AcSIR), CSIR-National Institute for Interdisciplinary Science and Technology (CSIR-NIIST), Trivandrum 695019, India
E-mail: ajayaghosh@niist.res.in

[b]Division of Chemistry and Biological Chemistry, School of Physical and Mathematical Sciences, Nanyang Technological University 21 Nanyang Link, 637371, Singapore.
School of Materials Science and Engineering, Nanyang Technological University, 639798, Singapore
E-mail: zhaoyanli@ntu.edu.sg

[c]School of Chemical and Biomedical Engineering, Nanyang Technological University, 62 Nanyang Drive, 637459, Singapore.

[d]Faculty of Materials and Energy, Southwest University, 2 Tiansheng Road, Beibei, Chongqing, 400715, People's Republic of China.

[e]Department of Nuclear medicine & PET, Singapore General Hospital, Outram Road, 169608, Singapore.

[f]School of Materials Science and Engineering, Nanyang Technological University, 639798, Singapore.

§These authors contributed equally to this work.

Table of Contents

1.	Synthesis and characterization	S3
2.	Supporting figures	S8
2.1.	Absorption and emission spectra of USq dye in DMSO	S8
2.2.	Absorption and emission spectra of USq upon addition of different concentrations of GSH	S9
2.3.	Sensitivity studies	S10
2.4.	Mechanism of fluorophore release	S11
2.5.	Reversibility studies	S12
2.6.	Fluorescence responses of USq dye with various amino acids	S13
2.7.	pH-Dependent stability and reactivity of USq dye	S13
2.8.	Cell viability test	S14

1. Synthesis and characterization

1.1. Scheme for the synthesis of the starting materials

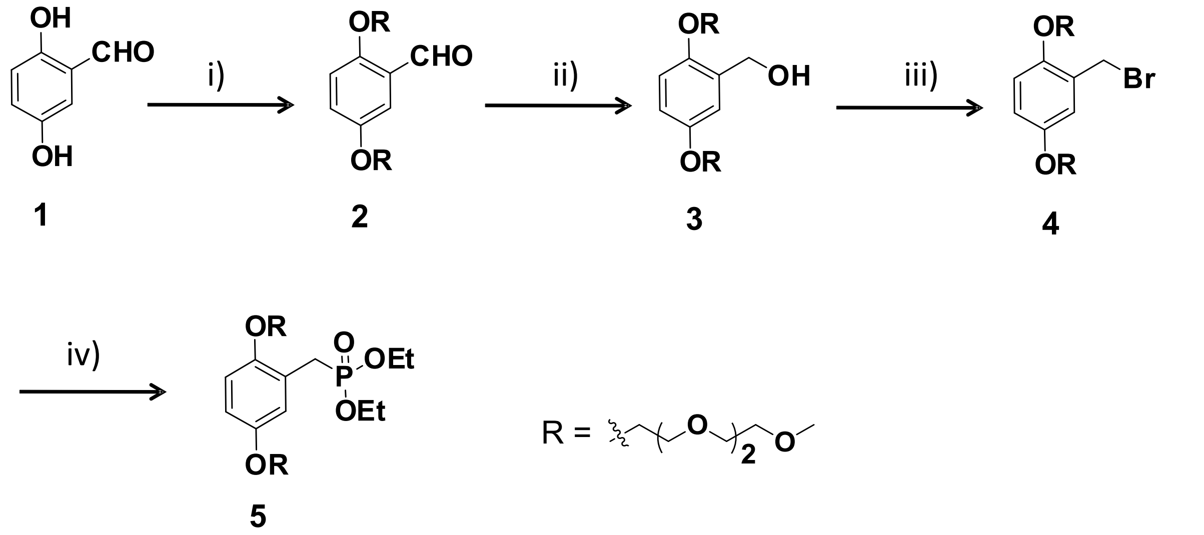

Scheme S1. Reagents and conditions: [i] 2-(2-(2-methoxyethoxy)ethyl 4-methylbenzenesulphonate, K_2CO_3, ACN, 60 °C, 24 h; [ii] $NaBH_4$, MeOH, DCM, rt, 3h; [iii] PBr_3, dry DCM, rt, 12 h; [iv] $P(OEt)_3$, 100 °C, 12 h.

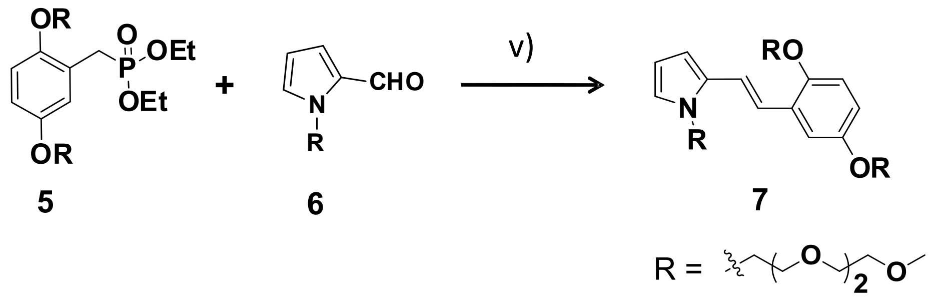

Scheme S2. Reagents and conditions: [v] NaH, dry THF, 70 °C, 12 h.

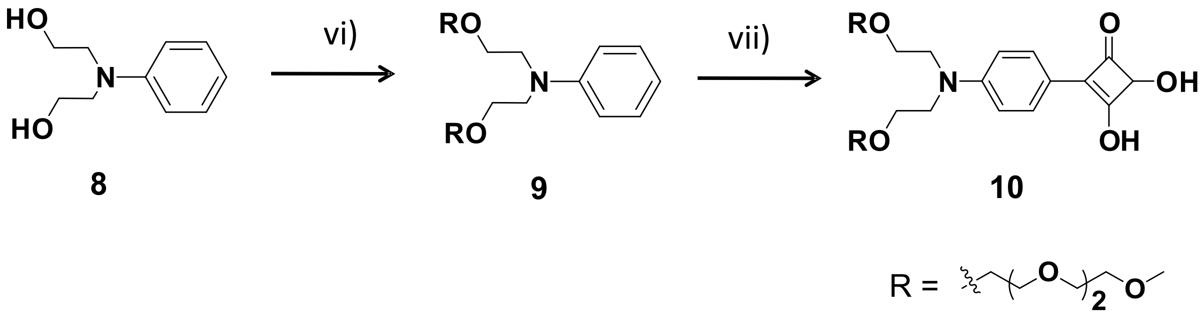

Scheme S3. Reagents and conditions: [vi] 2-(2-(2-methoxyethoxy)ethyl 4-methylbenzenesulphonate, KOH, THF, 70 °C, 24 h; [vii] squarylchloride, benzene, 80 °C , 6 h and CH_3COOH, HCl, 100 °C, 3 h.

1.2. Scheme for the synthesis of USq and SSq

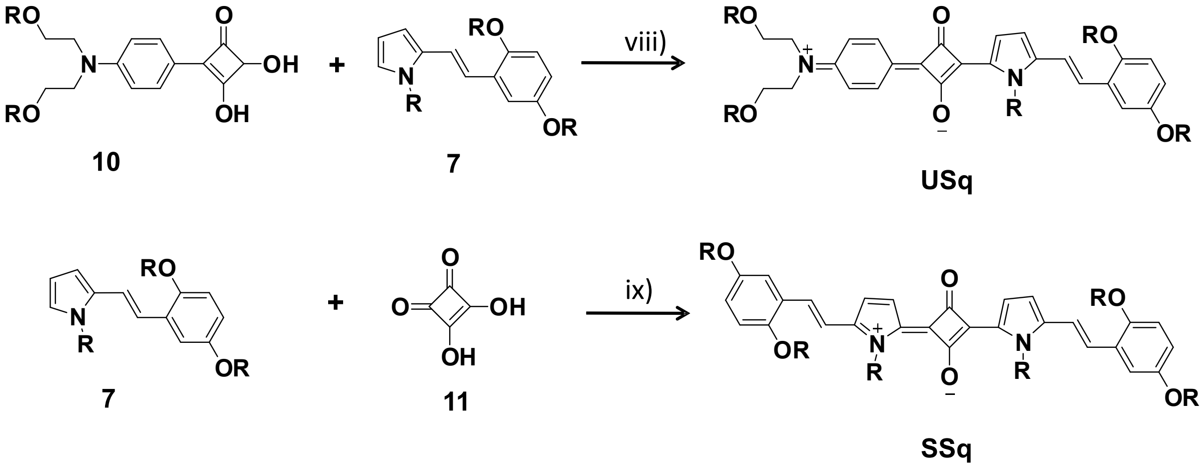

Scheme S4. Reagents and conditions: [viii] and [ix] 1:1 *n*-butanol/benzene azeotropic mixture, 90 °C, 10 h.

Synthesis of 2,5-bis(2-(2-(2-methoxyethoxy)ethoxy)ethoxy)benzaldehyde (2)

To a solution of 2,5-dihydroxybenzaldehyde (3.45 g, 25 mmol) and activated potassium carbonate (17.2 g, 125 mmol) in dry acetonitrile (50 mL) under argon atmosphere, triethylene glycol tosylate (20 g, 62.5 mmol) was added drop wise and the reaction mixture was refluxed for 48 h. The reaction mixture was cooled and the solvent was removed under reduced pressure. The residue obtained was then suspended in water and extracted with dichloromethane. The organic layer was separated, washed with brine, dried over anhydrous Na_2SO_4 and concentrated to give a crude product which was further purified by column chromatography over silica gel (100-200 mesh) using 1% methanol/$CHCl_3$ to give the desired product as a pink colored liquid. Yield 65%; ^{1}H NMR (500 MHz, $CDCl_3$, TMS) δ (ppm): 10.46 (s, 1H, *CHO*), 7.32 (s, 1H, Ar-*H*), 7.16 (d, 1H, Ar-*H*), 6.96 (d, 1H, Ar-*H*), 4.21 (t, 2H), 4.12 (t, 2H), 3.89-3.83 (t, 4H), 3.74-3.54 (m, 16H), 3.38-3.37 (s, 6H) ppm; ^{13}C NMR (125 MHz, $CDCl_3$) δ (ppm): 193.12, 156.60, 1562.91, 124.64, 120.82, 115.73, 114.60, 71.65, 70.38, 69.32, 59.31. HRMS (FAB) calcd for $C_{21}H_{34}O_9$ (M^+): 430.2, found: 431.6.

Synthesis of (2,5-bis(2-(2-(2-methoxyethoxy)ethoxy)ethoxy)phenyl)methanol (3)

To a solution of 2,5-diglycoxybenzaldehyde (7.74 g, 18 mmol) in dry DCM (100 mL) and methanol (5 mL), $NaBH_4$ (0.68 g, 18 mmol) was added in portion by keeping the temperature at 0 °C followed by stirring for 3 h under room temperature. The excess $NaBH_4$ was neutralized with ice and the solvents were removed by distillation under reduced pressure. The residue was extracted with dichloromethane, washed successively with water, brine and

dried over anhydrous Na_2SO_4. The combined organic layer was evaporated under reduced pressure to afford the desired product as a colorless liquid. Yield 94%; ^{1}H NMR (500 MHz, $CDCl_3$, TMS) δ (ppm): 6.89 (s, 1H, Ar-*H*), 6.81 (d, 2H, Ar-*H*), 4.61 (s, 2H), 4.32 (t, 2H), 4.29 (t, 2H), 3.79-3.81 (t, 4H), 3.70-3.52 (m, 16H), 3.38-3.37 (s, 6H); ^{13}C NMR (125 MHz, $CDCl_3$) δ (ppm): 149.12, 135.61, 114.01, 112.12, 71.60, 70.49,70.18, 60.52, 59.31. HRMS (FAB) calcd for $C_{21}H_{36}O_9$ (M^+): 432.51, found: 432.16.

Synthesis of 2-(bromomethyl)-1,4-bis(2-(2-(2-methoxyethoxy)ethoxy)ethoxy) benzene (4)

To a solution of 2,5-diglycoxybenzyl alcohol (6 g, 14 mmol) in dry dichloromethane (50 mL), PBr_3 (1.32 mL, 14 mmol) was added in drop wise by keeping the temperature at around 0 °C. After 12 h of stirring, the reaction mixture was poured into crushed ice and extracted with dichloromethane washed with brine and dried over anhydrous Na_2SO_4. The solvent was evaporated under reduced pressure to give benzyl bromide as the pure product. Yield 94%; ^{1}H NMR (500 MHz, $CDCl_3$, TMS) δ (ppm): 6.91 (s, 1H, Ar-*H*), 6.79 (d, 2H, Ar-*H*), 4.54 (s, 2H), 4.31 (t, 2H), 4.30 (t, 2H), 3.75-3.79 (t, 4H), 3.68-3.50 (m, 16H), 3.37-3.40 (s, 6H); ^{13}C NMR (125 MHz, $CDCl_3$) δ (ppm): 149.31, 147.12, 127.89, 115.21, 113.80, 112.21, 71.60, 70.42, 70.21, 69.63, 59.23, 29.71. HRMS (FAB) calcd for $C_{21}H_{35}BrO_8$ (M^+): 495.40, found: 496.81.

Preparation of diethyl 2,5-bis(2-(2-(2-methoxyethoxy)ethoxy)ethoxy)benzyl phosphonate (5)

2,5-Diglycoxybenzyl bromide (4.9 g, 10 mmol) was heated with triethyl phophite (2 mL) at 80-85 °C. After 12 h, the unreacted triethyl phophite was removed under reduced pressure resulting in a colorless liquid. Yield 98%; ^{1}H NMR (500 MHz, $CDCl_3$, TMS) δ (ppm): 6.92 (s, 1H, Ar-*H*), 6.73 (d, 2H, Ar-*H*), 4.22 (q, 4H), 4.21 (t, 2H), 4.12 (t, 2H), 3.89-3.83 (t, 4H), 3.73-3.54 (m, 16H), 3.37-3.38 (s, 6H) 3.26 (d, 2H), 1.27 (t, 6H); ^{13}C NMR (125 MHz, $CDCl_3$) δ (ppm): 152.59, 150.68, 121.34, 117.23, 113.84, 113.32, 71.64, 70.21, 67.67, 61.53, 58.63, 26.82, 17.77. HRMS (FAB) calcd for $C_{25}H_{45}O_{11}P$ (M^+): 552.59, found: 552.63.

Synthesis of (*E*)-2-(2,5-diglycoxystyryl)-1-glycol-1*H*-pyrrole (7)

A suspension of sodium hydride (720 mg, 30 mmol) in dry THF was slowly added to a solution of 2,5-diglycoxybenzyl phosphonate (2.87 g, 5.2 mmol) and *N*-glycol pyrrole-2-carboxaldehyde (**6**) (1.25 g, 5.2 mmol) in dry THF. After refluxing for 12 h, the reaction mixture was cooled and the THF was removed under reduced pressure. The residue obtained

was suspended in water and extracted with dichloromethane. The organic layer was separated, washed with brine, dried over anhydrous Na_2SO_4 and concentrated to give a crude product which was further purified by column chromatography over silica gel (100-200 mesh) using 3% methanol/dichloromethane. Yield 60%; ^{1}H NMR (500 MHz, $CDCl_3$, TMS) δ (ppm): 7.15 (d, J = 16.1 Hz, vinylic-*H*), 7.08 (d, J = 16.2 Hz, vinylic-*H*), 7.01 (d, 1H, Ar-*H*), 6.81 (d, 1H, Ar-*H*), 6.72 (t, 2H, Ar-*H*), 6.48 (d, 1H, Ar-*H*), 6.14 (t, 1H, Ar-*H*), 4.14 (t, 4H), 3.85 (t, 4H), 3.73-3.76 (t, 4H), 3.61-3.72 (m, 12H), 3.50-3.65 (m, 12H), 3.60 (s, 9H); ^{13}C NMR (125 MHz, $CDCl_3$) δ (ppm): 156.12, 146.70, 130.31, 127.90, 123.55, 119.65, 115.60, 114.91, 114.21, 111.39, 108.69, 72.64, 71.60, 70.41, 69.38, 59.31, 50.51. HRMS (FAB) calcd for $C_{33}H_{53}NO_{11}$ (M^+): 639.36, found: 638.27.

Preparation of *N*-phenyl-*N*-(2,5,8,11-tetraoxatridecan-13-yl)-2,5,8,11-tetraoxatridecan-13- amine (9)

A suspension of **8** (1.2 g, 6.62 mmol), triethylene glycol tosylate (4.5 g, 14.15 mmol) and potassium hydroxide (1.25 g, 22.30 mmol) in dry THF (100 mL) was refluxed for 24 h under an argon atmosphere. After removing the solvent under reduced pressure, water (50 mL) was added to hydrolyse the excess tosylate to the corresponding alcohol. After extracting with dichloromethane, the organic layer was dried over anhydrous Na_2SO_4 and concentrated to give a crude product, which was further purified by column chromatography over silica gel (100-200 mesh) using 2% methanol/ ethyl acetate to give the pure product as a pale-yellow oil. Yield 65%; ^{1}H NMR (500 MHz, $CDCl_3$, TMS) δ (ppm): 7.11 (t, 2H, Ar-*H*), 6.62 (d, 2H, Ar-*H*), 6.56 (t, 1H, Ar-*H*), 3.86 (t, 4H), 3.54-3.74 (m, 34H); ^{13}C NMR (125 MHz, $CDCl_3$) δ (ppm): 147.62, 129.72, 115.79, 71.78, 70.42, 70.36, 68.36, 61.43, 60.12. HRMS (FAB) calcd for $C_{24}H_{43}NO_8$ (M^+): 473.60, found: 474.61.

Preparation of 3-*N,N*-(diglycolamino)phenyl-4-hydroxy-3-cyclobutene-1,2-dione (10)

Squaryl chloride (600 mg, 4 mmol) and *N,N*-diglycol aniline **9** (1.9 g, 4 mmol) were dissolved in 50 mL dry benzene and refluxed for 6 h. After removing the solvent under reduced pressure, the crude product was purified by column chromatography over silica gel (100-200 mesh) using 2% methanol/dichloromethane to give a yellow-orange liquid. The residue was dissolved in a mixture of acetic acid (20 mL), hydrochloric acid (1 mL) and water (20 mL). This mixture was refluxed for 2 h, and cooled to room temperature. The solvent is removed under reduced pressure to give the pure product as a yellow liquid. Yield 60%; ^{1}H NMR (500 MHz, $CDCl_3$, TMS) δ (ppm): 8.00 (d, 2H, Ar-*H*), 7.10 (d, 2H, Ar-*H*),

3.76 (t, 4H), 3.52-3.76 (m, 34H); ^{13}C NMR (125 MHz, $CDCl_3$) δ (ppm): 190.51, 182.29, 148.80, 130.61, 122.71, 111.24, 96.68, 71.61, 70.45, 70.14, 70.13, 68.17, 59.32. HRMS (FAB) calcd for $C_{28}H_{45}NO_{11}$ (M^+): 571.66, found: 572.71.

General procedure for the syntheses of squaraine derivatives USq and SSq

The squaraine dye, USq was synthesized by condensing (*E*)-2-(2,5-diglycoxystyryl)-1-methyl-1H-pyrrole, (**7**) (572 mg, 1.0 mmol) and 3-*N*,*N*-(diglycolamino)phenyl-4-hydroxy-3-cyclobutene-1,2-dione (**10**) (655 mg, 1.0 mmol) in 1:1 *n*-butanol/benzene mixture (80 mL) under azeotropic conditions (Scheme S4). After refluxing for 10 h, the reaction mixture obtained was cooled followed by the removal of the solvents. The crude product was then precipitated from petroleum ether, filtered and redissolved in $CHCl_3$. The crude product obtained was purified by column chromatography over silica gel (100-200 mesh) using 4% methanol/dichloromethane. SSq was prepared starting from 7 and squaric acid in 2:1 ratio using similar procedures as in the case of USq. SSq was purified by column chromatography over silica gel (100-200 mesh) using 3% MeOH / $CHCl_3$.

USq: Yield 40%; ^1H NMR (500 MHz, $CDCl_3$, TMS) δ (ppm): 8.20 (d, 2H, Ar-*H*), 7.80 (d, 1H, Ar-*H*), 7.58 (d, *J* = 16.0 Hz, 1H, vinylic-*H*), 7.30 (d, *J* = 16.5 Hz, 1H, vinylic-*H*), 7.10 (s, 1H, Ar-*H*), 6.89 (d, 1H, Ar-*H*), 6.79 (d, 2H, Ar-*H*), 6.65 (d, 2H, Ar-*H*), 4.96 (t, 2H), 4.08 (m, 4H), 3.84 (t, 2H), 3.80 (t, 4H), 3.68 (m, 8H), 3.64 (m, 8H), 3.53-3.61 (m, 28H), 3.45-3.50 (m, 8H), 3.42 (t, 2H), 3.36 (t, 2H), 3.28-3.33 (m, 12H), 3.20 (s, 3H); ^{13}C NMR (125 MHz, $CDCl_3$) δ (ppm): 181.86, 178.21, 179.75, 172.56, 154.85, 153.47, 153.09, 148.12, 134.07, 130.61, 132.70, 128.15, 126.87, 120.47, 117.94, 117.11, 115.35, 113.16, 113.76, 73.12, 71.62, 70.40, 70.40, 70.15, 70.02, 66.64, 68.89, 53.35, 52.41, 48.46. MALDI-TOF-MS: calculated *m/z* for $C_{61}H_{94}N_2O_{21}$: 1191.40, found: 1192.31.

SSq: Yield 45%; ^1H NMR (500 MHz, $CDCl_3$, TMS) δ (ppm): 7.83 (d, 2H, Ar-*H*), 7.59 (d, *J* = 16.5 Hz, 2H, vinylic-*H*), 7.34 (d, *J* = 16.6 Hz, 2H, vinylic-*H*), 7.16 (s, 2H, Ar-*H*), 6.92 (d, 1H, Ar-*H*), 6.86 (d, 4H, Ar-*H*), 4.97 (t, 4H), 4.16 (m, 8H), 3.91 (t, 6H), 3.86 (t, 3H), 3.77 (m, 8H), 3.64-3.72 (m, 16H), 3.48-3.59 (m, 16H), 3.45 (m, 4H), 3.32-3.40 (m, 16H); ^{13}C NMR (125 MHz, $CDCl_3$) δ (ppm): 153.27, 151.44, 148.31, 129.91, 126.91, 126.80, 117.02, 116.05, 114.25, 113.83, 113.07, 71.93, 71.91, 71.82, 71.03, 70.81, 70.70, 70.66, 70.58, 70.49, 69.83, 69.79, 69.00, 68.07, 59.03, 58.86, 46.92. MALDI-TOF-MS: calculated *m/z* for $C_{70}H_{104}N_2O_{24}$: 1357.57, found: 1359.08.

2. Supporting figures

2.1. Absorption and emission spectra of USq dye in DMSO

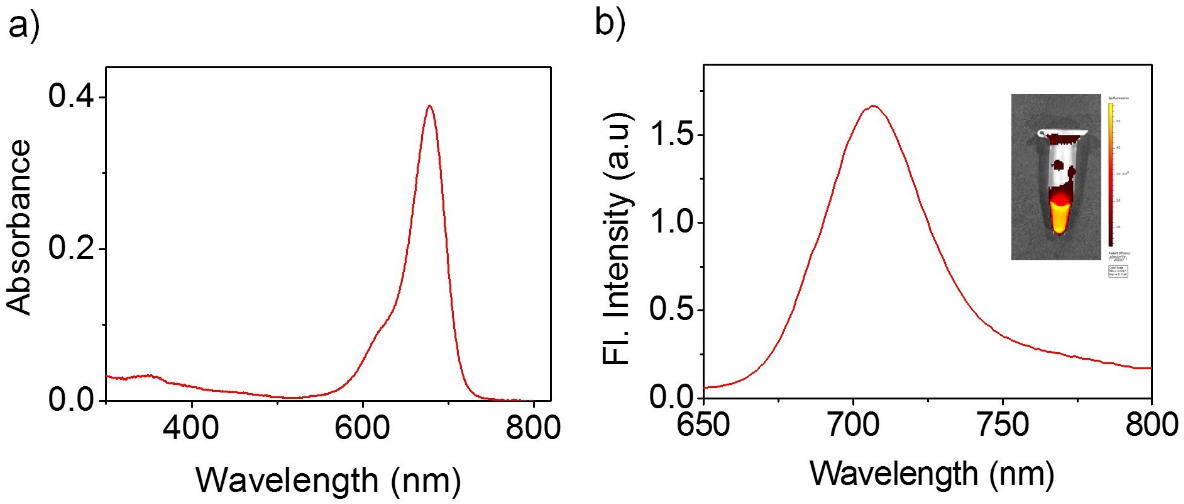

Figure S1. (a) UV/Vis absorption and (b) emission spectra (λ_{ex} @ 640 nm) of USq (2 μM) in DMSO. Inset shows false color pixel intensity map of fluorescence from USq upon excitation at 640 nm.

2.2. Absorption and emission spectra of USq upon addition of different concentrations of GSH

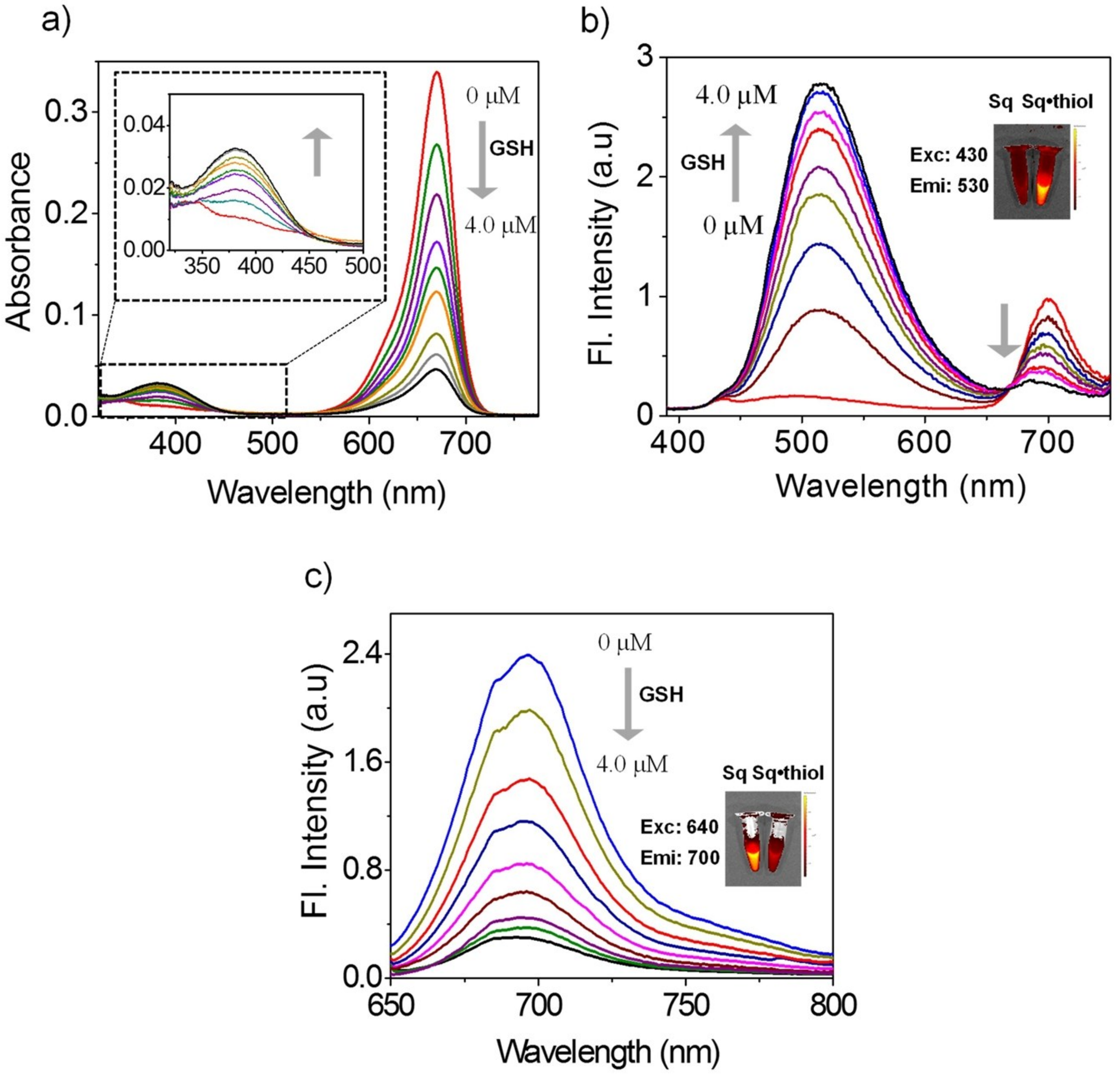

Figure S2. (a) UV/Vis absorption, as well as (b) and (c) fluorescence responses of USq (2 μM, in 96% phosphate buffer pH 7.8/DMSO) upon the addition of GSH (0-4 μM) (λ_{ex} = 380 nm for (b) and 640 nm for (c)). Inset of (b) and (c) shows the fluorescence of USq as false-color pixel intensity at 700 and 530 nm in an eppendorf before and after the addition of GSH under illumination using 430 and 640 nm respectively.

2.3. Sensitivity studies

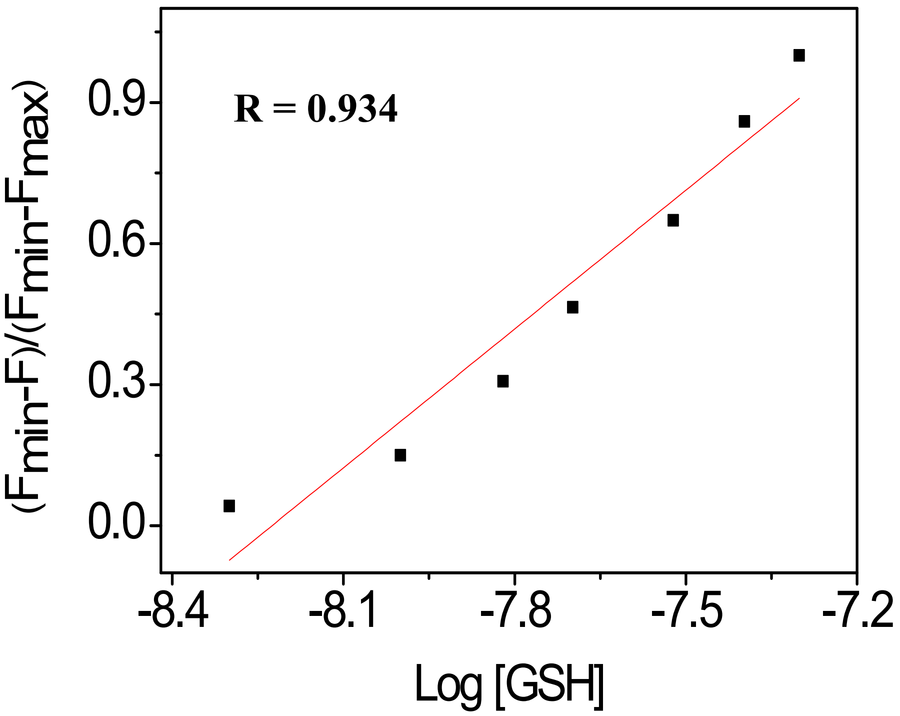

Figure S3. Fluorescence intensity at 520 nm (λ_{ex} @ 380 nm) of USq (2 µM, 96% phosphate buffer, pH 7.8-DMSO) at each concentration of GSH added, normalized between the minimum fluorescence intensity, found at zero equiv. of GSH, and the maximum fluorescence intensity, found at [GSH] = 1 × 10^{-7} M. Each point was acquired 10 min after exposure of GSH.

2.4. Mechanism of fluorophore release

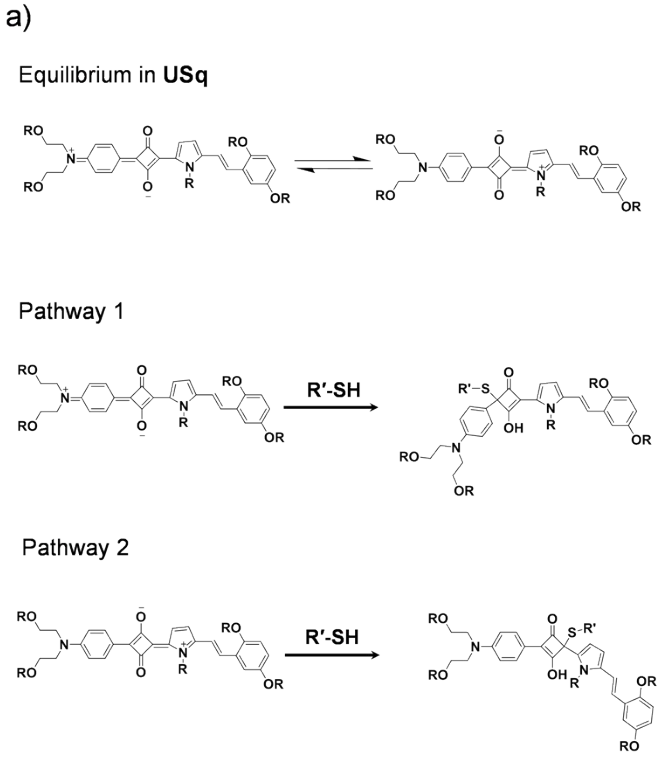

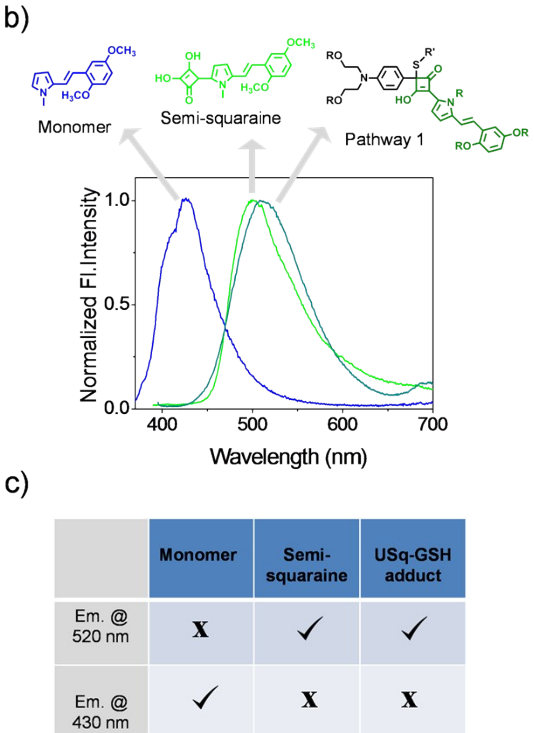

c)

	Monomer	Semi-squaraine	USq-GSH adduct
Em. @ 520 nm	x	✓	✓
Em. @ 430 nm	✓	x	x

Figure S4. (a) Possible pathways for USq to undergo nucleophilic addition reaction with GSH. (b) Comparison of fluorescence emission spectrum for USq-GSH adduct (dark green line), styryl-pyrrole moiety (blue line) and semi-squaraine unit (green line) in 50% phosphate buffer (pH 7.8)/DMSO mixture. (c) Table showing summary of spectroscopic evidence for thiol attacking site *via* the comparison of different emission spectra.

2.5. Reversibility studies

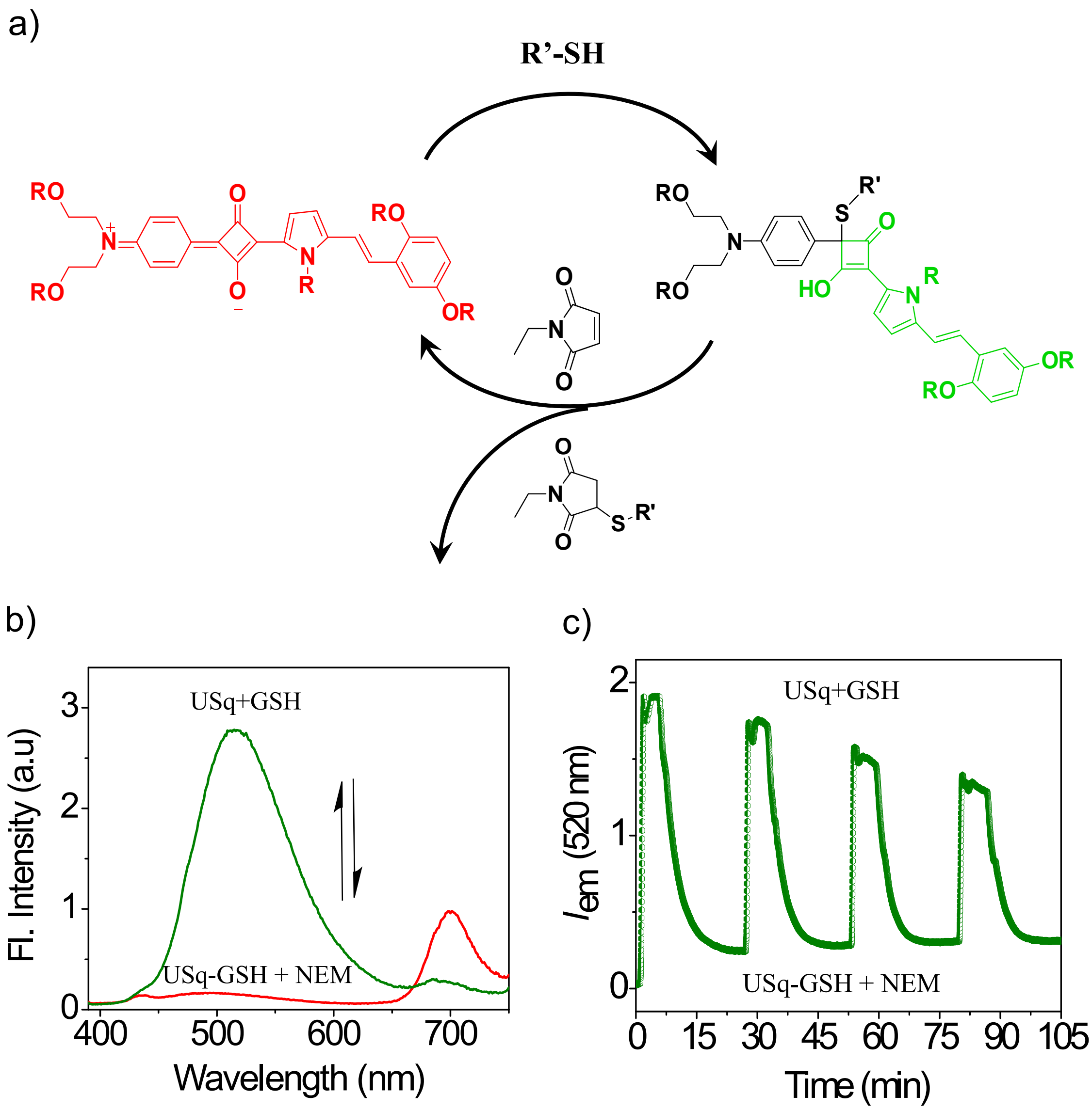

Figure S5. a) Schematic representation for the reversible interaction of USq with GSH. b) Fluorescence response of USq-GSH adduct in the presence and absence of NEM. c) Time dependent fluorescence responses of USq at 520 nm with alternative addition of GSH and NEM. These experiments were performed in 96% phosphate buffer, pH 7.8-DMSO with 2 μM USq, 4 μM GSH and 8 μM NEM (λ_{ex} @ 380 nm).

2.6. Fluorescence responses of USq dye with various amino acids

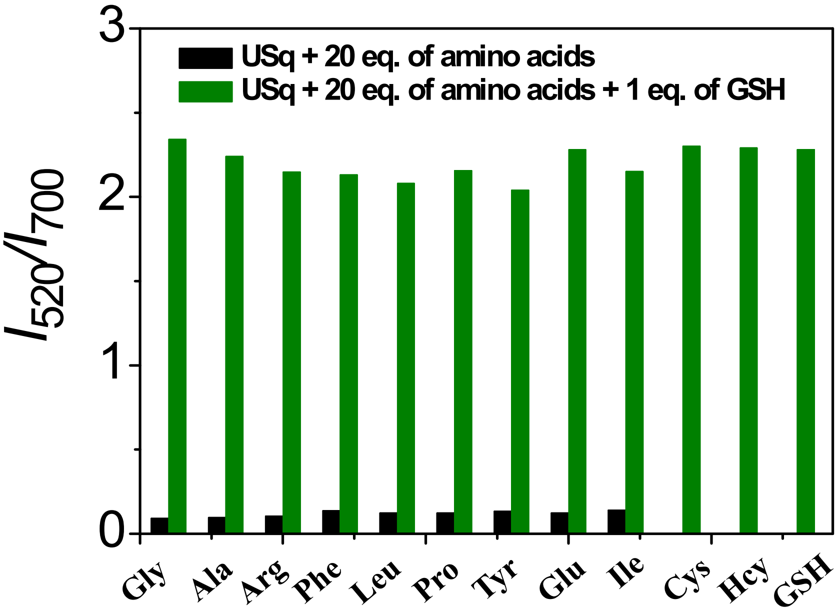

Figure S6. Fluorescence responses of USq (2 μM, 96% phosphate buffer, pH 7.8-DMSO, λ_{ex} @ 380 nm) towards various amino acids (AA) in the presence and absence of GSH. [AA] = 40 μM, [Cys] = 2 μM, [Hcy] = 2 μM, [GSH] = 2 μM.

2.7. pH-Dependent stability and reactivity of USq dye

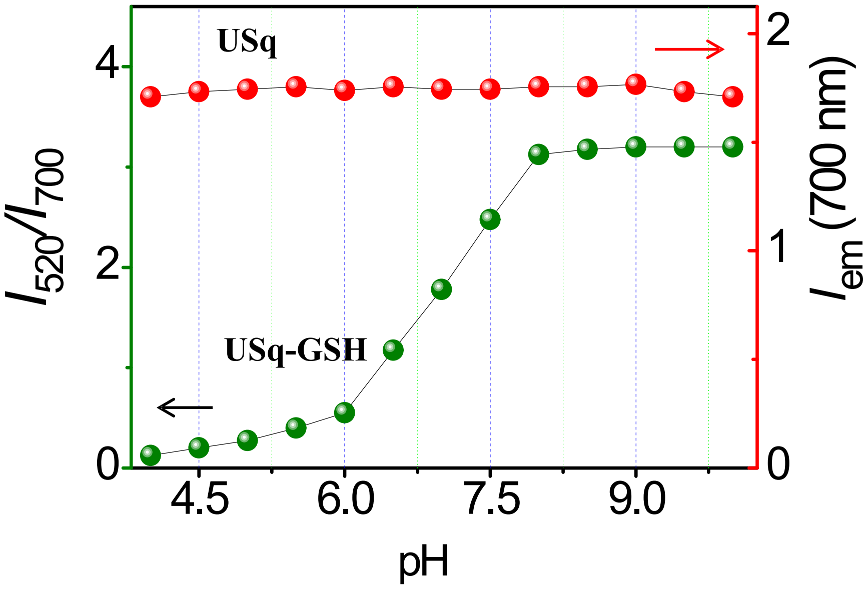

Figure S7. Fluorescence responses of USq (2μM, 96% phosphate buffer, pH 7.8-DMSO) with (λ_{ex} @ 380 nm) and without (λ_{ex} @ 640 nm) GSH (4 μM) as a function of pH. Each point was acquired 10 min after exposure (in the case of GSH) at 37 °C.

2.8. Cell viability test

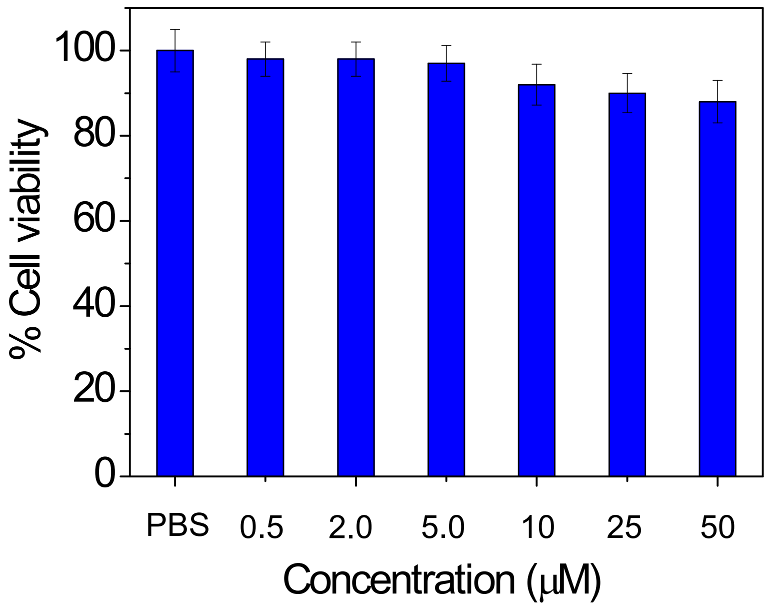

Figure S8. Cytotoxicity of USq in Huh-7 cells evaluated by MTT assay. The cells were incubated with USq of different concentrations (0-50 μM) for 48 h.

Inherent cytotoxicity of USq was evaluated using the MTT (3-(4,5-dimethylthiozolyl-2)-2,5-diphenyltetrazolium bromide) viability assay with human cervical cancer cell lines (Huh-7 cell lines) incubated for 48 h. The cell viability was estimated by varying the concentrations of USq in micromolar range. Results obtained from viability assay are shown in Figure S7. The results clearly indicate that USq has low cytotoxicity at low to moderate concentrations. The low cytotoxicity and good solubility of USq in aqueous conditions with excellent monomeric properties further support its usage as a potential probe for *in vitro* or *in vivo* monitoring of thiols.
